# Long-Term Bone Density Changes and Fracture Risk in Myasthenia Gravis: Implications for FRAX^®^ Tool Application

**DOI:** 10.3390/healthcare12171793

**Published:** 2024-09-08

**Authors:** Shingo Konno, Takafumi Uchi, Hideo Kihara, Hideki Sugimoto

**Affiliations:** Department of Neurology, Toho University Ohashi Medical Center, Tokyo 153-8515, Japan; takafumi.uchi@med.toho-u.ac.jp (T.U.); hideo.kihara@med.toho-u.ac.jp (H.K.); sugi-h@oha.toho-u.ac.jp (H.S.)

**Keywords:** myasthenia gravis, glucocorticoid, osteoporosis, osteoporotic fracture, bone mineral density, fracture risk assessment tool (FRAX^®^)

## Abstract

Myasthenia gravis (MG) patients often require long-term glucocorticoid therapy, which may affect bone health. This study aimed to assess long-term changes in bone mineral density (BMD), evaluate osteoporotic fracture incidence, and examine the relationship between MG-specific factors and bone health outcomes over a 10-year period. This single-center, prospective cohort study included 28 MG patients. BMD, T-scores, Z-scores, and bone turnover markers were measured at baseline. FRAX^®^ scores were calculated and adjusted for glucocorticoid dose. Fracture occurrence was monitored for over 10 years. Five (17.9%) patients experienced major osteoporotic fractures during follow-up. The fracture group had significantly lower baseline BMD and T-scores than the no-fracture group. Baseline FRAX^®^ scores for major osteoporotic fracture risk were significantly higher in the fracture group (median 19.0% vs. 5.7%, *p* = 0.001). The fracture group progressed from osteopenia at baseline to osteoporosis by the end of this study. This study highlights the importance of early and regular bone health assessments in MG patients, particularly those receiving long-term glucocorticoid therapy. The FRAX^®^ tool may be valuable for fracture risk stratification in this population. These findings can inform clinical practice and improve long-term management strategies for MG patients who are at risk of osteoporotic fractures.

## 1. Introduction

Myasthenia gravis (MG) is an autoimmune disorder characterized by muscle weakness and fatigue that primarily affects the neuromuscular junction through the production of autoantibodies against acetylcholine receptor antibodies (AChR-Abs) and muscle-specific kinase antibodies (MuSK-Abs) [1].

The prevalence of MG has been increasing globally, with a particularly notable in-crease in the elderly population in Japan. A recent nationwide survey revealed a prevalence of 23.1 per 100,000 individuals in Japan, highlighting a growing public health concern [2].

The management of MG often involves long-term glucocorticoid therapy, which, while effective in controlling the disease, poses a significant risk to bone health. Prolonged glucocorticoid use is associated with reduced bone mineral density (BMD) and an elevated risk of osteoporotic fractures, presenting a critical challenge in the long-term care of patients with MG [3,4]. Wakata et al. highlighted the potential for osteoporosis in patients with MG receiving high-dose prednisolone but found a lower than expected incidence of osteoporosis in their study population [3]. Konno et al. further investigated this issue and demonstrated that glucocorticoid-induced osteoporosis significantly affects the quality of life of patients with MG [5]. However, recent research has shown that bisphosphonates and vitamin D therapy can maintain bone density in patients with MG at two-year intervals [6].

The Fracture Risk Assessment Tool (FRAX^®^) has emerged as a valuable instrument for estimating fracture risk in the general population [7]. Recent findings now demonstrate the efficacy of the FRAX^®^ tool in accurately predicting fracture risks in patients with MG [8]. The study revealed that higher FRAX^®^ scores are significantly associated with increased fracture risk, affirming the tool’s critical role in the clinical management of patients with MG to enhance treatment outcomes and patient care. These insights call for the integration of FRAX^®^ assessments into routine clinical evaluations to provide a strategic approach for managing fracture risks tailored specifically to the needs of patients with MG.

Given the chronic nature of MG and the prolonged use of glucocorticoids in its treatment, there is a pressing need to understand the long-term implications on bone health in these patients [9]. Moreover, validating the effectiveness of the FRAX^®^ tool in this specific patient group could significantly enhance risk assessment and guide preventive strategies [10].

This study aimed to address these knowledge gaps by (1) assessing long-term changes in BMD among patients with MG over a 10-year period, (2) evaluating the incidence of osteoporotic fractures in this cohort, and (3) examining the relationship between MG-specific factors, including treatment regimens, and bone health outcomes.

By providing comprehensive data on bone health trajectories and fracture risk in patients with MG, this study seeks to inform clinical practice and improve long-term management strategies for this vulnerable population.

## 2. Materials and Method

### 2.1. Study Design and Participants

**Figure 1:**
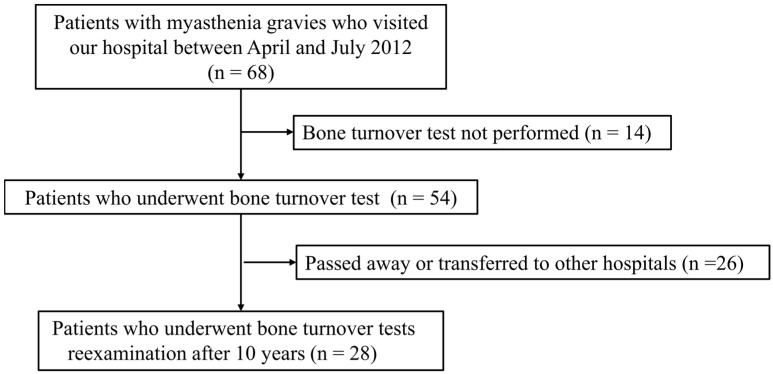
Flow diagram of patient selection.

This single-center, prospective cohort study was conducted at the Toho University Ohashi Medical Center. Between April and July 2012, 68 patients with MG visited our hospital. The FRAX^®^ score was calculated for 54 patients who underwent BMD testing and, 10 years later, BMD was reexamined in 28 patients, including 23 who had not experienced fractures (no-fracture group) and 5 who developed fractures (Figure 1).

Between April and July 2012, 68 patients with myasthenia gravis visited our hospital. Bone turnover tests were performed on 54 patients, whereas 14 patients did not undergo these tests. Over the 10-year follow-up period, 30 patients were lost to follow-up due to death or transfer to other hospitals. The final analysis included 28 patients who underwent bone turnover test reexamination after 10 years.

MG diagnosis is based on medical history, signs of muscle weakness during daily activities, positive tests for AChR-Ab and MuSK-Ab, decreased complex muscle action potential voltage during repetitive nerve stimulation tests, and special tests, such as the ice pack test or edrophonium test [10]. Symptom severity was assessed using the MG Foundation of America (MGFA) clinical classification, MG Activity of Daily Living, Quantitative MG (QMG) score, and MGFA postintervention status [11,12].

### 2.2. BMD Measurement and T-Scores and Z-Scores Calculation

BMD was measured in the femoral neck using a dual-energy X-ray absorptiometry system (GE Healthcare, Chicago, IL, USA) following standard procedures to ensure image consistency [13]. T-scores were calculated using the following formula: T-score = (patient’s BMD − average BMD of a young adult)/(standard deviation of BMD in young adults). For the Z-score calculation, we used the following formula: Z-score = (patient’s BMD − average BMD of the age-matched population)/(standard deviation of BMD of the age-matched population) [14].

### 2.3. Test for Bone Turnover Markers in Blood

Serum levels of the bone isoform of alkaline phosphatase (BAP) and pyridinoline cross-linked N-terminal telopeptides of type I collagen (NTx) were measured using a chemiluminescent enzyme immunoassay to assess the bone turnover balance [15].

### 2.4. Calculation of FRAX^®^ Score with BMD

The FRAX^®^ tool estimates fracture risk considering factors such as age, sex, body weight, previous fractures, family history of hip fractures, smoking, heavy alcohol (≥3 units per day) use, long-term use of certain glucocorticoids (>5 mg prednisone or equivalent per day for ≥3 months), rheumatoid arthritis, and other diseases that can cause secondary osteoporosis (e.g., secondary hyperparathyroidism, diabetes, chronic kidney disease) [16]. FRAX^®^ scores were calculated in three ways: with BMD, without BMD, and adjusted for glucocorticoid dose. Previous fractures, particularly in the thoracic and lumbar spine, were primarily assessed through patient self-reports rather than direct radiographic evidence [17].

### 2.5. FRAX^®^ Score Adjustment Based on Glucocorticoid Dose

The FRAX^®^ scores were adjusted based on glucocorticoid dosage: 0.8× for prednisolone use of <2.5 mg/day, 1.0× for 2.5–7.5 mg/day, and 1.15× for ≥7.5 mg/day [18].

### 2.6. Data Collection on Outcomes

The primary outcome measure was the occurrence of major osteoporotic fractures between June 2012 and June 2022. Data were obtained from an initial cohort study that began in 2012. The participants provided written consent for the original study, and the secondary use of these data was publicly announced on the hospital website.

### 2.7. Statistical Analysis

Descriptive data were summarized as number, prevalence, or median (interquartile range [IQR] [25%, 75%]). A Shapiro–Wilk test was used to assess normality. Between-group comparisons were performed using the Mann–Whitney U test, χ2 test, and Fisher’s exact test.

Statistical analyses were performed using R software (version 3.2.2; R Foundation for Statistical Computing, Vienna, Austria) with the “rms” package [19].

## 3. Results

### 3.1. Study Design and Participants

This study included 28 patients with MG (left column in Table 1), comprising 24 females (85.7%) and 4 males (14.3%). The median age of patients at baseline was 62.0 years [IQR: 48.7–65.2], with a median MG onset age of 41.0 years [IQR: 29.2–50.5] and a median disease duration of 11.5 years [IQR: 4.0–25.5]. The distribution of MG subtypes was as follows: 9 patients (32.0%) with ocular MG (oMG), 13 patients (46.4%) with generalized early-onset MG (gEOMG), 1 patient (3.5%) with generalized late-onset MG (gLOMG), 4 patients (14.0%) with generalized thymoma-associated MG (gTAMG), and 2 patients (7.1%) with antibody-negative MG. According to the MGFA classification, 9 patients (32.1%) were in Class I, 9 (32.1%) in Class II, 3 (10.7%) in Class III, 5 (17.9%) in Class IV, and 2 (7.1%) in Class V. The MG-specific measures revealed the following median scores: an MG ADL score of 1.0 [IQR: 1.0–3.2], a QMG score of 3.5 [IQR: 2.0–6.2], an MG composite score of 2.5 [IQR: 0.0–5.0], and an MG quality of life 15 score of 9.5 [IQR: 2.0–24.0]. At baseline, no significant differences were found in these scores, nor in the MGFA classification or MGFA postintervention status between the fracture and no-fracture groups. Regarding treatment, 21 patients (75.0%) were using prednisolone, with a median dose of 2.0 mg/day [IQR: 0.0–4.0] at baseline, while 10 patients (35.7%) were using calcineurin inhibitors. Treatment for MG-related factors, such as prednisolone use, maximum dose, treatment duration, and total dose within a 1-year period, did not show significant differences between the fracture and no-fracture groups at baseline. 

### 3.2. BMD Measurements and T-Scores/Z-Scores

As presented in Table 2, at baseline, the majority of patients were female (85.7% overall), with all patients in the fracture group being female (100%) compared to 82.6% in the no-fracture group. Hip BMD was lower in the fracture group compared to the no-fracture group, although this difference did not reach statistical significance (fracture group: median 0.6 [IQR: 0.5–0.7] vs. no-fracture group: 0.8 [IQR: 0.7–0.9], *p* = 0.070). Similarly, T-scores were lower in the fracture group, but the difference was not statistically significant (fracture group: median −2.1 [IQR: −2.9 to −0.9] vs. no-fracture group: −1.0 [IQR: −1.4 to 0.1], *p* = 0.097). Z-scores showed no significant difference between the groups (fracture group: 0.3 [IQR: −0.3 to 1.5] vs. no-fracture group: 0.8 [IQR: 0.6 to 1.4], *p* = 0.313).

After 10 years, the fracture group showed significantly lower hip BMD than the no-fracture group (fracture group: 0.5 [IQR: 0.5, 0.5] vs. no-fracture group: 0.6 [IQR: 0.6, 0.7], *p* = 0.005). T-scores were also significantly lower in the fracture group (fracture group: −2.5 [IQR: −2.6, −2.5] vs. no-fracture group: −1.0 [IQR: −1.4, −0.2], *p* = 0.006). Although Z-scores showed no statistically significant difference between the groups at 10 years, there was a trend towards lower values in the fracture group (fracture group: −0.5 [IQR: −1.0, −0.4] vs. no-fracture group: 0.1 [IQR: −0.7, 1.3], *p* = 0.171).

### 3.3. Bone Turnover Markers

As presented in Table 2, at baseline, derum BAP levels showed no significant difference between the fracture and no-fracture groups (fracture group: median 12.0 μg/L [IQR: 8.9, 12.6] vs. no-fracture group: 10.0 μg/L [IQR: 7.0, 12.1], *p* = 0.515). Similarly, serum NTx levels at baseline did not differ significantly between the groups (fracture group: 13.4 nmol BCE/L [IQR: 12.0, 14.0] vs. no-fracture group: 12.5 nmol BCE/L [IQR: 10.1, 15.5], *p* = 0.569).

After 10 years, serum BAP levels remained similar between the groups, although, compared to baseline values, there was a slight increase in both the groups (fracture group: 12.0 μg/L [IQR: 12.0, 15.0] vs. no-fracture group: 10.0 μg/L [IQR: 8.0, 12.5], *p* = 0.103). While both groups showed some increase in BAP levels over the 10-year period, the fracture group demonstrated a slightly larger increase, particularly in the upper quartile range. However, this difference did not reach statistical significance.

### 3.4. FRAX^®^ Score Calculations

Table 2 demonstrates significant differences in baseline FRAX^®^ scores. Major osteoporotic fracture risk with BMD was significantly higher in the fracture group (median 19.0% [IQR: 19.0–31.0]) than in the no-fracture group (5.7% [IQR: 3.2–8.8], *p* = 0.001).

### 3.5. FRAX^®^ Score of Original Version and Adjustments for Glucocorticoid Dose

The baseline FRAX^®^ scores for major osteoporotic fracture risk with BMD showed significant differences between the fracture and no-fracture groups. The fracture group demonstrated a substantially higher risk compared to the no-fracture group (median 19.0% [IQR: 19.0–31.0] vs. 5.7% [IQR: 3.2–8.8], *p* = 0.001). When adjusted for glucocorticoid dose, the FRAX^®^ scores maintained a significant difference between the groups. The adjusted scores for major osteoporotic fracture risk with BMD remained higher in the fracture group compared to the no-fracture group (median 15.2% [IQR: 15.2–31.0] vs. 4.8% [IQR: 2.8–7.9], *p* = 0.002). These results indicate that both the original and glucocorticoid-adjusted FRAX^®^ scores were significantly higher in the fracture group at baseline.

### 3.6. Outcome Data Collection

During the 10-year follow-up period, five patients (17.9%) experienced major osteoporotic fractures. The distribution of the fracture types is presented in Table 2. Vertebral fractures were the most common, with two patients (40% of fracture cases) experiencing lumbar vertebral fractures and one patient (20%) having a thoracic vertebral fracture.

## 4. Discussion

This 10-year prospective study initially included 68 patients with MG, of whom 54 underwent bone metabolism tests at baseline. At the 10-year follow-up point, 28 patients were available for reassessment. Of these 28 patients, five (17.9%) experienced major osteoporotic fractures during the follow-up period. Our findings revealed two critical aspects. (1) Patients who experienced a fracture during the follow-up period had significantly higher FRAX^®^ scores than those who did not, despite having similar baseline BMD and T-scores. This finding supports previous research indicating that FRAX^®^ can provide additional fracture risk information beyond BMD alone [16]. (2) The fracture group progressed from osteopenia at baseline to osteoporosis by the end of the study period, suggesting that the rate of bone density loss over time may be a crucial factor for fracture risk in patients with MG [20].

Although the baseline hip BMD and T-scores were not significantly different between the fracture and no-fracture groups, these differences became more pronounced and statistically significant after 10 years. This highlights the importance of long-term monitoring of bone health in patients with MG.

While FRAX^®^ scores were calculated for all 54 patients at baseline, our analysis focused on 28 patients who were available for the 10-year follow-up. This analysis revealed significant differences in FRAX^®^ scores between the fracture and no-fracture groups. The fracture group had significantly higher baseline FRAX^®^ scores for major osteoporotic fracture risk. This finding supports the potential utility of the FRAX^®^ tool in identifying high-risk patients, even when baseline BMD and T-scores are not significantly different [21,22].

The observed bone loss and increased fracture risk are likely multifactorial, with long-term glucocorticoid use being the primary concern [5]. Glucocorticoids impair bone metabolism by suppressing osteoblast function and enhancing osteoclast survival [23]. However, other factors specific to MG or its management may also contribute to bone health outcomes, underlining the complex interplay among the disease, its treatment, and skeletal integrity.

The use of bone metabolism medications did not differ significantly between the fracture and no-fracture groups. Bisphosphonates were used in 60% and 54.5% of patients in the fracture and no-fracture groups, respectively This suggests that, despite receiving standard osteoporosis treatment, some patients still experience fractures, highlighting the potential limitations of current therapeutic approaches in this population [24].

The choice of bone metabolism medication in this study was influenced by the clinical guidelines and therapeutic options available in Japan at the time of the initiation of this study in 2012. While the American College of Rheumatology guidelines suggested the use of denosumab and teriparatide for patients with high FRAX^®^ scores [25], the Japanese Guidelines for the Management and Treatment of Steroid-Induced Osteoporosis (revised in 2014) primarily recommended bisphosphonates as first-line agents [26]. This difference in guidelines and the availability of newer treatments in Japan during the study period may explain the low use of teriparatide and denosumab in the high-FRAX^®^ group.

The Japanese guidelines published in 2023 recommended five drugs (oral or injectable bisphosphonates, anti-receptor activator of nuclear factor kappa-Β ligand antibodies, teriparatide, eldecalcitol, or selective estrogen receptor modulators) as a result of a systematic review and other factors as treatment options increased. In addition, while alendronate and bisphosphonate were previously specified as first-line agents, they are no longer specified as first-line agents in this revision. The reason for this is supplemented by the fact that “there were no trials that directly compared each drug in a rigorous manner” [27].

It is worth noting that recent advances in molecular targeted therapies for MG, such as eculizumab and efgartigimod, may potentially reduce the reliance on long-term glucocorticoid use [28,29]. These novel treatments target specific pathways involved in autoimmune responses, potentially offering better disease control with fewer side effects. As these therapies become more widely adopted, they may contribute to improved bone health outcomes in patients with MG by reducing cumulative glucocorticoid exposure. Future studies should investigate the long-term effects of these targeted therapies on bone health and fracture risk in patients with MG. These results emphasize the need for personalized approaches to bone health management in patients with MG. Regular assessment of fracture risk using tools like FRAX^®^, monitoring of BMD, and early implementation of preventive strategies should be considered as part of standard care for patients with MG, especially those on long-term glucocorticoid therapy [30].

This study had several limitations. First, the number of patients was small, and this study involved only one institution. Therefore, the results cannot be generalized to patients at other facilities or from different demographics.

Despite these limitations, a notable strength of this study is its long follow-up period of 10 years in a specific category of patients with myasthenia gravis. This extended observation period allows for a comprehensive assessment of long-term bone health outcomes and fracture risks in this patient population, which is relatively rare in the literature. The focus on MG patients provides valuable insights into the bone health trajectories of individuals with this specific neuromuscular disorder, contributing to a better understanding of the long-term skeletal effects of both the disease and its treatments.

Furthermore, the use of standardized measures, such as FRAX^®^ scores and regular BMD assessments, enhances the reliability and clinical relevance of the findings. This longitudinal approach offers a unique perspective on the evolution of fracture risk in MG patients over time, which can inform clinical practice and future research directions.

This decade-long prospective study sheds new light on the complex relationship among MG, its treatment, and long-term bone health outcomes. Our findings underscore the superior predictive value of FRAX^®^ scores over baseline BMD and T-scores in assessing fracture risk among patients with MG. This revelation challenges the current approaches to bone health management in this population and highlights the need for a paradigm shift in clinical practice.

The results of this study call for a more nuanced and personalized approach to maintaining bone health in patients with MG. They suggest that clinicians should look beyond traditional bone density measurements and incorporate comprehensive risk assessment tools, like FRAX^®^, from the early stages of MG diagnosis and treatment. Furthermore, the observed progression from osteopenia to osteoporosis in the fracture group emphasizes the dynamic nature of bone health in patients with MG and the necessity for continuous monitoring and intervention.

Our findings also raise important questions regarding the efficacy of the current osteoporosis treatments in the context of MG. The similar use of bone metabolism medications between the fracture and no-fracture groups suggests that standard therapies may not be sufficiently protective for all patients with MG, particularly those with high FRAX^®^ scores. It is noteworthy that the final years of this study coincided with the COVID-19 pandemic, which emerged in 2019 and may have significantly impacted bone mineral density (BMD) and fracture risk. Pandemic-related lifestyle changes have been shown to negatively affect bone health across various populations, including younger adults [31]. SARS-CoV-2 infection can potentially influence bone health through direct effects on bone marrow cells, increased inflammation, alterations in bone turnover markers, and indirect effects such as reduced physical activity, nutritional deficiencies, and increased corticosteroid use [32,33].

For MG patients who are already at risk for bone loss due to their condition and treatments, these pandemic-related factors may have exacerbated their vulnerability to osteoporosis and fractures. While this study was not specifically designed to address the impact of the COVID-19 pandemic, it is possible that the observed bone density changes and fracture risks were influenced by these additional factors. Future research should consider the long-term effects of major global events, like pandemics, on bone health outcomes in chronic disease populations. This underscores the importance of maintaining bone health strategies, even during periods of reduced mobility or healthcare access, especially for patients with conditions, like MG, who are already at increased risk for bone loss.

## 5. Conclusions

This 10-year prospective study of patients with MG reveals significant long-term impacts on bone health, particularly in those undergoing glucocorticoid therapy. Our findings demonstrate that 17.9% of MG patients experienced major osteoporotic fractures over the follow-up period, with the fracture group progressing from osteopenia at baseline to osteoporosis by this study’s end. Notably, baseline FRAX^®^ scores showed superior predictive value for fracture risk compared to initial BMD and T-scores alone, even when adjusted for glucocorticoid dose.

Despite similar use of bone metabolism medications between fracture and non-fracture groups, significant differences in bone health outcomes were observed. This suggests that current standard therapies may not be sufficiently protective for all MG patients, particularly those with high FRAX^®^ scores.

These results highlight the need for early and regular bone health assessments in MG patients, especially those receiving long-term glucocorticoid therapy. The FRAX^®^ tool appears to be valuable for fracture risk stratification in this population, potentially guiding more targeted preventive strategies and treatment approaches.

## Figures and Tables

**Table 1:** Patient characteristics and treatments for myasthenia gravis.

Factor	Overall	Fracture Group	No-Fracture Group	*p*
	28	5	23	
Follow-up period (days)	4018 [4003, 4027]	4024 [4014, 4024]	4018 [3999, 4027]	0.617
Onset age of MG (years)	41.0 [29.2, 50.5]	53.0 [39.0, 71.0]	39.0 [27.0, 48.5]	0.077
Disease duration (years)	11.5 [4.0, 25.5]	13.0 [5.0, 27.0]	10.0 [4.0, 24.0]	0.857
Subtype (n, %)				
oMG	9 (32.0)	2 (40.0)	7(22.7)	0.428
gEOMG	13 (46.4)	1 (20.0)	12 (54.5)	
gLOMG	1 (3.5)	0 (0.0)	1 (4.5)	
gTAMG	4 (14.0)	2 (40.0)	2 (9.1)	
Ab-negative MG	2 (7.0)	0 (0.0)	2 (9.1)	
MGFA classification (n, %)				
Class I	9 (32.1)	2 (40.0)	7 (30.4)	0.149
Class II	9 (32.1)	0 (0.0)	9 (39.1)	
Class III	3 (10.7)	0 (0.0)	3 (13.0)	
Class VI	5 (17.9)	2 (40.0)	3 (13.0)	
Class VI	2 (7.1)	1 (20.0)	1 (4.3)	
MGFA-PIS (n, %)				
Complete stable remission	3 (10.7)	1 (20.0)	2 (8.7)	1.000
Pharmacological remission	2 (7.1)	0 (0.0)	2 (8.7)	
Improved	13 (46.4)	3 (60.0)	10 (43.5)	
Minimal manifestations	7 (25.0)	1 (20.0)	6 (26.1)	
Unchanged	3 (10.7)	0 (0.0)	3 (13.0)	
MG ADL (point)	1.0 [1.0, 3.2]	3.0 [1.0, 5.0]	1.0 [1.0, 3.0]	0.370
QMG score (point)	3.5 [2.0, 6.2]	4.0 [2.0, 6.0]	3.0 [2.0, 6.5]	0.928
MG composite (point)	2.5 [0.0, 5.0]	2.0 [0.0, 4.0]	3.0 [0.0, 5.0]	0.926
MG quality of life 15 (point)	9.5 [2.0, 24.0]	13.0 [3.0, 33.0]	8.0 [2.0, 21.5]	0.696
Treatment for MG				
Prednisolone use (n, %)	21 (75.0)	3 (60.0)	18 (78.3)	0.574
Max dose of PSL (mg/day)	50.0 [35.0, 50.0]	50.0 [50.0, 55.0]	42.5 [35.0, 50.0]	0.060
Duration of PSL treatment (years)	9.0 [2.8, 13.0]	10.0 [7.4, 11.5]	7.3 [2.7, 18.2]	0.687
Total PSL dose within a 1-year period (mg)	916 [0.00, 2592]	954 [687, 1121]	905 [0.0, 3044]	0.937
PSL dose at baseline (mg/day)	2.0 [0.0, 4.0]	2.0 [0.0, 2.5]	2.0 [0.0, 5.2]	0.768
PSL dose 10 years later (mg/day)	0.0 [0.0, 2.5]	0.0 [0.0, 0.0]	0.0 [0.0, 2.5]	0.500
Calcineurin inhibitors use (n, %)	10 (35.7)	3 (60.0)	7 (30.4)	0.315
Dose of tacrolimus (mg/day)	3.0 [0.7, 3.0]	1.5 [0.7, 2.2]	3.0 [2.2, 3.2]	0.453
Dose of cyclosporine (mg/kg/day)	2.5 [2.4, 4.5]	4.5 [4.5, 4.5]	2.4 [2.3, 3.0]	0.277

Note: Data presentation: n (%); median (interquartile range IQR [25%, 75%]). Abbreviations: o-MG, ocular myasthenia gravis; g–EOMG, generalized early-onset MG; g–LOMG, generalized late-onset MG; g–SNMG, generalized seronegative MG; g–TAMG, generalized thymoma associated MG, MGFA, Myasthenia Gravis Foundation of America; PIS, postintervention status; ADL, activity of living; QMG, quantitative MG score; PSL, prednisolone; NA; not applicable.

**Table 2:** Parameters related to bone turnover and FRAX^®^ Score.

Factor	Overall	Fracture Group	No-Fracture Group	*p*
	28	5	23	
Follow-up period (days)	4018 [4002, 4026]	4024 [4014, 4024]	4018 [4001, 4027]	0.528
Until fracture development (days)		2437 [2158, 2929]	—	
Medication of bone turnover (n, %)				
Bisphosphates	16(57.1)	3 (60,0)	13 (54.5)	0.331
Vitamin D therapy	0 (0.0)	0 (0.0)	0 (0.0)	NA
Teriparatide (induced after fracture)	2 (7.0)	2(40.0)		
Serum BAP at baseline (μg/L)	10.0 [7.1, 12.2]	12.0 [8.9, 12.6]	10.0 [7.0, 12.1]	0.515
Serum BAP 10 years later (μg/L)	10.0 [8.7, 13.0]	12.0 [12.0, 15.0]	10.0 [8.0, 12.5]	0.103
Serum NTx at baseline (nmol BCE/L)	13.3 [10.4, 16.6]	13.4 [12.0, 14.0]	12.5 [10.1, 15.5]	0.569
Serum NTx 10 years later (nmol BCE/L)	Not measured			
BMD of hip at baseline	0.7 [0.7, 0.9]	0.6 [0.5, 0.7]	0.8 [0.7, 0.9]	0.070
BMD of hip 10 years later	0.6 [0.6, 0.7]	0.5 [0.5, 0.5]	0.6 [0.6, 0.7]	0.005
T-score at baseline	−1.0 [−1.5, −0.2]	−2.1 [−2.9, −0.9]	−1.0 [−1.4, 0.1]	0.097
T-score 10 years later	−1.1 [−1.7, −0.6]	−2.5 [−2.6, −2.5]	−1.0 [−1.4, −0.2]	0.006
Z-score at baseline	0.8 [0.3, 1.4]	0.3 [−0.3, 1.5]	0.8 [0.6, 1.4]	0.313
Z-score 10 years later	0.1 [−0.7, 1.0]	−0.5 [−1.0, −0.4]	0.1 [−0.7, 1.3]	0.171
Factors of FRAX^®^ calculation				
Age	62.0 [48.7, 65.2]	66.0 [66.0, 75.0]	58.0 [47.0, 63.5]	0.005
Sex, female (%)	24 (85.7)	5 (100.0)	19 (82.6)	1.000
Body mass index (kg/m^2^)	21.6 [20.5, 23.7]	21.5 [20.8, 22.2]	21.7 [20.3, 24.0]	0.904
Previous fracture (n, %)	0 (0.0)	0 (0.0)	0 (0.0)	NA
Parent’s hip fracture (n, %)	2 (7.1)	2 (40.0)	0 (0.0)	0.026
Current smoker (n, %)	2 (7.1)	0 (0.0)	2 (8.7)	1000
Alcohol intake (>3 units/day) (n, %)	0 (0.0)	0 (0.0)	0 (0.0)	NA
Glucocorticoid use (>5 mg/day of PSL or equivalent for >3 months) (n, %)	20 (71.4)	3 (60.0)	17 (73.9)	0.606
Rheumatoid arthritis (n, %)	1 (3.6)	0 (0.0)	1 (4.3)	1.000
Diseases associated with secondary osteoporosis (n, %)	0 (0.0)	0 (0.0)	0 (0.0)	NA
Major osteoporotic fracture risk with BMD (%)	7.3 [3.6, 12.0]	19.0 [19.0, 31.0]	5.7 [3.2, 8.8]	0.001
Adjusted major osteoporotic fracture risk with BMD (%)	6.1 [3.6, 9.6]	15.2 [15.2, 31.0]	4.8 [2.8, 7.9]	0.002
Osteoporotic fractures within 10 years				
Hip fracture (n, %)		1 (20.0)		
Lumbar vertebra fracture (n, %)		2(40.0)		
Thoracic vertebra fracture (n, %)		1 (20.0)		
Proximal humerus fracture (n, %)		1 (20.0)		

Note: Data presentation: n (%); median (interquartile range IQR [25%, 75%]), BAP, alkaline phosphatase; NTx, pyridinoline cross-linked amino-terminal telopeptide of type I collagen; BMD, bone mineral density, NA; not applicable.

## Data Availability

The data are not publicly available because the information contained could compromise the privacy of the patients. The data supporting the findings of this study are available, except for the patients’ personal information, upon request from the corresponding author, Shingo Konno.
